# Objective Assessment of Medication Adherence Through Automated Pill Detection Using a Computer Vision Framework: Development and Evaluation Study

**DOI:** 10.2196/92528

**Published:** 2026-08-18

**Authors:** Haozhe Liu, Yaguang Zheng, Bei Wu, Yu Gan

**Affiliations:** 1 Department of Biomedical Engineering, Stevens Institute of Technology, Hoboken, NJ, United States; 2 Rory Meyers College of Nursing, New York University, New York, NY, United States; 3 Faculty of Arts and Science, New York University Shanghai, Shanghai, China; 4 Fischell Department of Bioengineering, University of Maryland, 3102 A. James Clark Hall, College Park, MD, 20742, United States, 1 2012165593; 5 Artificial Intelligence Interdisciplinary Institute at Maryland, University of Maryland, , College Park, MD, United States

**Keywords:** medication adherence, pill detection, pill counting, computer vision, digital health, Object Detection, Image Segmentation

## Abstract

**Background:**

Medication adherence remains a significant concern in both clinical practice and public health. Nonadherence to prescribed medication regimens is associated with poorer health outcomes, higher rates of hospitalization, and increased financial burdens on health care systems. Despite its critical role in ensuring treatment efficacy, adherence assessment is still largely dependent on patient self-reports, pharmacy refill records, or caregiver observations—methods that are often subjective, inconsistent, and unreliable. Therefore, there is an urgent need for objective, automated solutions to accurately monitor medication, particularly the information on the pills taken and intake behavior. AI techniques, especially computer vision, are promising solutions; however, their application in assessing medication adherence has several key pitfalls in maintaining robustness under environmental variation that limit their reliability and real-world applicability.

**Objective:**

This study aimed to develop a reliable and accurate computer vision framework for pill detection and counting using mobile phone images captured under diverse real-world conditions to support medication adherence assessment.

**Methods:**

This study is carried out using a public dataset on pill detection (N=152) and a curated dataset (N=60) on medication adherence assessment. Inspired by mixture of experts (MoE), we propose and implement a robust pill detection framework that integrates two expert-level object detection models, You Only Look Once (YOLOv12) and Faster Region-based Convolutional Neural Network (Faster R-CNN), with a Segment Anything Model (SAM). The pill counting performance was evaluated using mean absolute error (MAE) and accuracy. We further conduct a pilot study on a curated dataset that mimics a subject taking medicine for a period of 20 days. We evaluate sensitivity and specificity in medication adherence assessment.

**Results:**

The proposed multimodel framework substantially reduces both error types, achieving a low MAE of 0.167 on the Kaggle test set in pill counting and an accuracy of 0.933 in pill detection. In medication adherence simulation, the proposed framework achieves high accuracy (0.917), sensitivity (0.933), and specificity (0.900), significantly outperforming the evaluated YOLOv12 and Faster R-CNN baselines (*P*<.05). Performance remained robust on the self-collected dataset in medication-taking scenarios in the settings of household, restaurant, office, and outdoor environments.

**Conclusions:**

The proposed framework achieved high performance in pill counting, detection, and simulated medication-adherence classification. This proof-of-concept study has demonstrated the potential to enhance medication adherence among patients and support more reliable treatment monitoring.

## Introduction

### Background

Medication adherence refers to the degree individuals follow prescribed medication regimens in terms of timing, dosage, and frequency [1]. Adherence to the treatment regimen is a critical determinant of treatment effectiveness and long-term health outcomes [2]. Medication nonadherence remains a significant concern in clinical practice and public health, particularly for patients with chronic conditions such as diabetes, hypertension, and cardiovascular disease [3-5]. Nonadherence to prescribed medication regimens contributes to poorer health outcomes, increased rates of hospitalization, and substantial financial burdens on both patients and the health care system [6,7]. Prior studies have estimated that nonadherence to prescribed medications is associated with approximately 125,000 preventable deaths annually and contributes to US $100‐300 billion when both direct and indirect costs are included [8].

Given the critical role adherence plays in treatment efficacy, accurately assessing medication adherence is essential. However, current monitoring approaches are largely based on patient self-reports, pharmacy refill data, or caregiver observation [9]. These methods are often unreliable, subjective, and prone to recall bias [10,11]. This challenge is further intensified by the growing number of older adults with cognitive impairment, who may have even greater difficulty managing complex medication regimens [12]. As a result, there is an urgent need for objective, automated solutions to assess medication intake behavior accurately.

### AI in Medication Adherence

AI is a promising approach to objectively assessing medication adherence. Several studies have applied machine learning methods (eg, supervised learning, deep learning) to classify adherence status using self-reported adherence questionnaires [13-15] but such approaches remain highly dependent on self-reported input and can therefore be dramatically affected by reporting inaccuracies. Recently, computer vision models that analyze image- and video-based data provided by patients, such as photographs or short recordings of medications, have gained increasing attention. These models are particularly relevant in the management of noncommunicable diseases, such as cancer, cardiovascular disease, chronic respiratory disease, and diabetes [16]. By automatically detecting and counting pills, identifying medication containers, or tracking medication-taking behaviors over time, these approaches attempt to provide a more objective and scalable means of adherence verification [17,18]. Among those tasks, pill detection and counting is one of the most critical tasks, as it reflects the dose of the medicine that is taken. Compared with patient-reported methods, vision-based deep learning techniques reduce reliance on patient self-reporting, offering the potential for more accurate, timely, and continuous assessment of medication adherence in real-world settings.

Presently, researchers have identified several key pitfalls of AI-based pill counting applications that limit their reliability and real-world usability [19]. These challenges include sensitivity to lighting variation, background clutter, pill overlap and orientations, among others. Such limitations can lead to inaccurate pill counts and reduce the effectiveness of vision-based approaches for medication adherence assessment in unconstrained real-world settings.

### Goal of This Study

To address these limitations, this study aims to develop a reliable and accurate computer vision framework for pill detection and counting under diverse real-world imaging conditions to support objective medication adherence assessment. Specifically, we evaluate whether a multimodel design that integrates complementary object detectors, segmentation-based refinement, redundancy removal, and geometric validation can improve pill-counting accuracy and dose-level adherence classification compared with individual detector baselines.

### Related Work

#### Medication Adherence Monitoring Technologies

A range of technologies has been developed to monitor medication adherence, including self-reported questionnaires, pill counts, pharmacy refill records, caregiver observations, and electronic medication monitoring devices [9-11,19,20]. Although useful in clinical practice and research, many of these approaches provide only indirect or proxy measurements of medication-taking behavior; for example, self-reports are affected by recall and social-desirability bias, and pharmacy refill records confirm acquisition rather than correct dosing. Electronic monitoring systems, smart pill bottles, and smart medication adherence products can log container-opening events, reminders, dispensing records, or alerts [19,21] but these signals do not verify that the correct number of pills was removed, counted, or ingested. Existing adherence monitoring methods therefore remain limited when the goal is to directly assess dose-level medication intake.

#### AI-Based Pill Identification, Detection, and Counting

Computer vision has been widely studied for pill identification, detection, and verification. Public challenges and deep learning methods have enabled matching of consumer pill images to reference images and improved pill identification from mobile or consumer-quality photographs [22-24]. These studies demonstrate the feasibility of AI-based pill image analysis but most target pill identity recognition rather than dose-level adherence assessment. Other work has addressed pill localization, verification, or counting in more structured settings, such as hospital pharmacy workflows, device-based pill verification, and general pill-counting applications [19,25,26]. While relevant to pill detection and medication safety, daily-life pill counting for dose-level adherence assessment remains underexplored. In this study, we focus on whether the visible pill count in patient-captured still images matches the prescribed dosage under realistic daily conditions.

## Methods

### Development of Computer Vision Models

**Figure 1.**
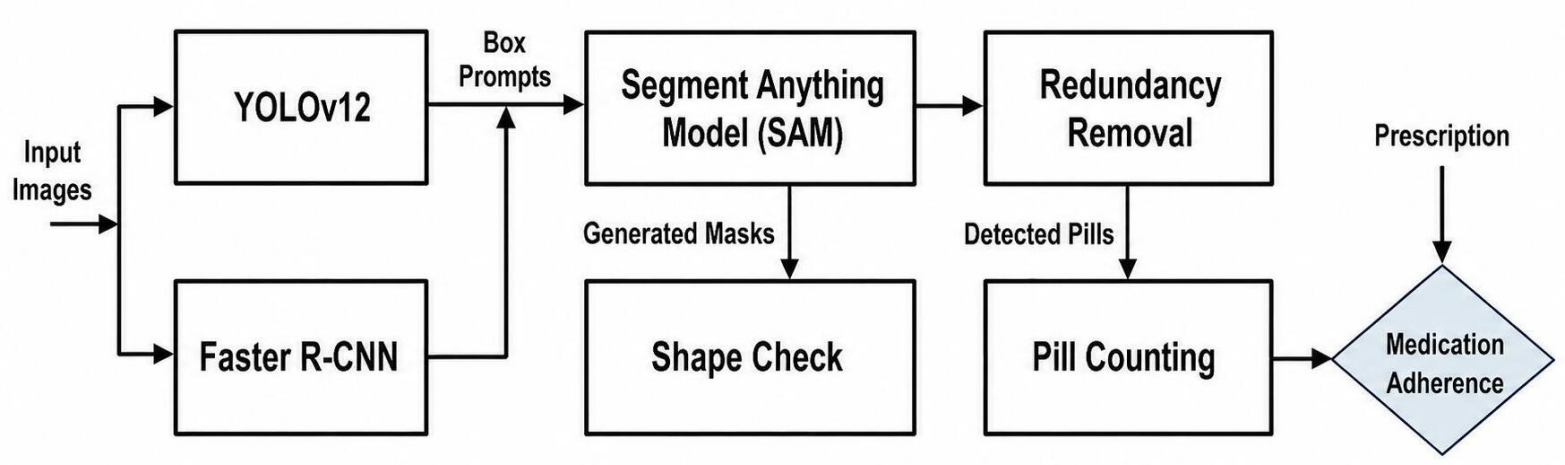
Overview of the proposed framework for medication adherence. Faster R-CNN: Faster Region-based Convolutional Neural Network; YOLOv12: You Only Look Once version 12.

We develop a multimodel framework that integrates two well-established object detection models and one segmentation model, inspired by the concept of Mixture of Experts (MoE) [27]. As shown in Figure 1, three models You Only Look Once (YOLOv12) [28], Faster Region-based Convolutional Neural Network (Faster R-CNN) [29], and Segment Anything Model (SAM) [30] serve as three experts for decision making. Two stages are involved, where the first stage roughly identifies the region of interest within the human hand, and the second stage refines pill counts within the candidate regions via pixel-wise segmentation. In the first stage, two individual detectors aim to reduce reliance on a single detection paradigm and improve coverage across heterogeneous visual patterns. In the second stage, a detection-guided segmentation strategy leverages the generalization capability of SAM, a foundation model trained on large-scale real-world image data, to enable robust segmentation across diverse imaging conditions without requiring task-specific training. This modular design supports reliable performance in complex real-world environments and facilitates adaptation to different medication-taking scenarios. Potential regions of interest identified in the first stage are used as prompts to generate pill masks, which substantially reduces the computational cost of SAM while enabling efficient and accurate segmentation. This mask generation step enables accurate delineation of object boundaries and contributes to robust pill detection under complex real-world photographing conditions while supporting real-time inference. Those two stages are optimized using redundant detection removal after object detection and shape smoothness check after pill segmentation. If the intersection over union (IoU) between two segmentation masks exceeded 0.8, the corresponding bounding box detections are considered to represent the same pill instance and are merged into a single detection, and the mask with the higher confidence score and greater geometric plausibility is retained. To further suppress false positives caused by background artifacts, a shape smoothness check module is developed to confirm the segmented region follows the round shape of a medical pill.

Specifically, YOLOv12 and Faster R-CNN are applied in parallel and independently to each input image, and their candidate bounding boxes are combined into a unified candidate region set rather than being processed sequentially. Each candidate box is then used as a prompt for SAM to generate a corresponding segmentation mask, and disagreements between the two detectors are resolved after segmentation through the mask-level redundancy removal and geometric shape validation described above, which prevents the same pill from being counted multiple times. This detector-prompted SAM strategy enables the framework to refine candidate pill regions at the pixel level while preserving the complementary detection patterns of the one-stage and two-stage detectors.

### Dataset Collection

We use a public dataset to evaluate the performance of pill detection and counting and a self-collected dataset to evaluate the performance of medication adherence assessment. A total of 152 images were obtained from a publicly available Kaggle dataset [31]. The dataset is randomly split into 10:2:3 for training, validation, and testing. We further evaluate our framework in a longitudinal medication adherence scenario. Specifically, the medication adherence scenarios cover four representative environment settings (ie, home, office, restaurant, and outdoor). The curated longitudinal medication adherence dataset included 60 images simulating a 20-day medication-taking scenario with three doses per day. The prescribed dose was four pills per image. Among these images, 30 were adherent cases with exactly four visible pills, and 30 were non-adherent cases with pill counts differing from the prescribed dose, including 18 under-dosing cases (1‐3 pills), 1 missed-dose case (0 pills), and 11 over-dosing cases (5 or more pills). The non-adherent cases had true pill counts of 0 (n=1), 1 (n=4), 2 (n=9), 3 (n=5), 5 (n=5), 6 (n=2), 7 (n=1), 8 (n=1), 9 (n=1), and 10 (n=1). This distribution was used to evaluate whether the framework could distinguish correct dosing from under-dosing and over-dosing errors.

### Evaluation Metrics

We directly evaluate the performance of pill counting and detection at both pill level and dosage (ie, image) level. At pill level, we evaluate the pill counting performance using mean absolute error (MAE), defined as the absolute difference between predicted and reference pill counts. At dosage level, we evaluate the accuracy on whether the entire image can be correctly identified or not. At both levels, we compare performance with the conventional AI models, Faster R-CNN and YOLOv12. For longitudinal study, we focus on assessing adherence vs non-adherence. Specifically, we evaluate sensitivity and specificity. Sensitivity is measured as the percentage of times correctly identifying adherence. In contrast, specificity is measured as the percentage of times correctly identifying non-adherence (eg, if one takes five pills rather than the required dosage of four, our AI model can identify that the number of pills is five, then it is correctly classified as non-adherence). For both evaluations, the ground truth is human annotation on number of pills in the image.

### Statistical Analysis

Because the proposed framework and the baseline models (YOLOv12 and Faster R-CNN) were evaluated on the same images, paired statistical tests were used, and all comparisons were performed separately for the public Kaggle pill-detection dataset and the longitudinal medication-adherence dataset. For dosage-level (image-level) accuracy, the exact McNemar test was applied to the paired correct and incorrect classification outcomes of the proposed framework and each baseline, so that only discordant image pairs, those classified correctly by one method but not the other, contributed to the test statistic; because the number of discordant image pairs was small, the exact binomial form of the McNemar test was used rather than the *χ*^2^ approximation. For pill counting, the per-image absolute counting error, defined as the absolute difference between the predicted and reference pill counts, was compared between the proposed framework and each baseline using the Wilcoxon signed-rank test, a non-parametric paired test that does not assume normally distributed differences. The same paired-testing procedure was applied to the adherence classification outcomes in the longitudinal medication-adherence dataset. All tests were two-sided, and statistical significance was defined as *P*<.05.

### Ethical Considerations

This study used a publicly available pill image dataset and a curated simulated medication-adherence dataset created for prototype evaluation. Ethics approval was not required because the study did not involve patient participants, identifiable individuals, protected health information, clinical records, or real medication-use behavior. The curated images were collected only to simulate pill-counting and dose-level adherence scenarios for technical evaluation of the proposed computer vision framework.

## Results

### Overall Performance in Pill Detection and Pill Counting

The proposed multi-model framework achieves a dosage-level accuracy of 0.933 with a pill-level MAE of 0.167, as shown in Table 1. Compared with single-model baselines on the same image set, Faster R-CNN achieved an accuracy of 0.733 with an MAE of 0.300, while YOLOv12 achieved an accuracy of 0.767 with the same MAE of 0.300. The proposed pill detection framework achieved higher accuracy than the comparison models. Specifically, it significantly outperformed Faster R-CNN, with McNemar test showing (*P*=.02) and the Wilcoxon signed-rank test showing (*P*=.03). It also significantly outperformed YOLOv12, with McNemar test showing (*P*=.02) and the Wilcoxon signed-rank test showing (*P*=.03). Since all reported *P* values were below .05, these results indicate that the performance improvements of the proposed framework over both baseline models were statistically significant. The highest accuracy and lowest MAE obtained from our framework indicates a superior performance over existing single AI models in pill counting. We also conducted an ablation study to evaluate the contribution of each component, as shown in Table 1. The individual detectors achieved an MAE of 0.300 and dosage-level accuracies of 0.733‐0.767. Combining the two detectors with SAM-based refinement and mask-level redundancy removal reduced the MAE to 0.200 and increased the accuracy to 0.900, and adding geometric shape validation in the full framework further reduced the MAE to 0.167 and increased the accuracy to 0.933.

**Table 1.** Pill detection performance and ablation study on the Kaggle test set, reporting pill-counting MAE^a^ and dosage-level accuracy.

Method	SAM^b^ refinement	Redundancy removal	Shape check	MAE	Accuracy
YOLOv12^c^	No	No	No	0.300	0.767
Faster R-CNN^d^	No	No	No	0.300	0.733
YOLOv12 & Faster R-CNN+ SAM + Redundancy removal	Yes	Yes	No	0.200	0.900
Full proposed method	Yes	Yes	Yes	0.167	0.933

a MAE: mean absolute error.

b SAM: segment anything model.

c YOLOv12: You Only Look Once version 12.

d R-CNN: region-based convolutional neural network.

**Figure 2.**
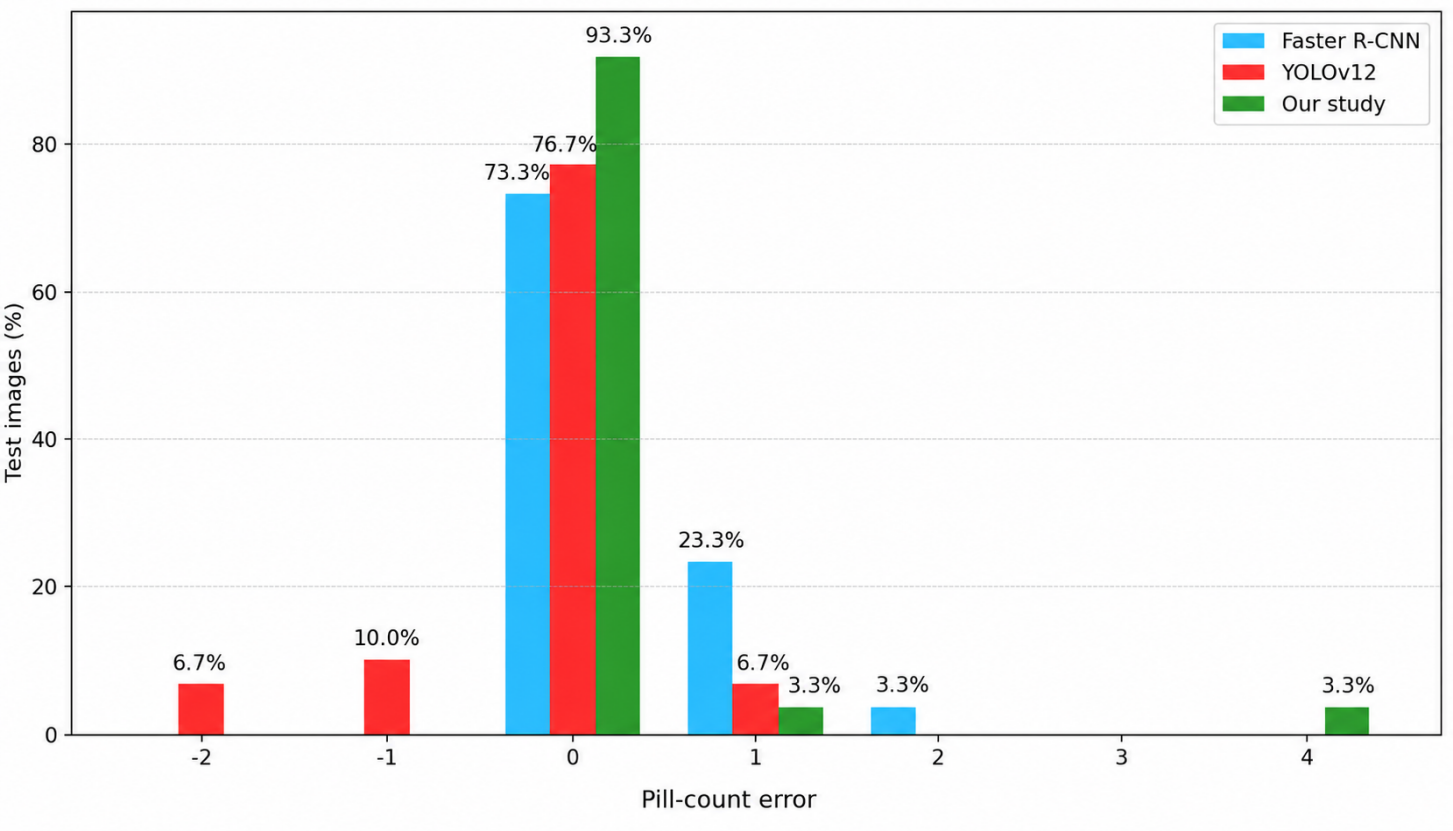
Evaluation of the proposed method’s performance compared to Faster R-CNN and YOLOv12 on the test dataset. Faster R-CNN: Faster Region-based Convolutional Neural Network; YOLOv12: You Only Look Once version 12.

The detailed distribution of pill-count errors for Faster R-CNN, YOLOv12, and the proposed method on the test dataset is listed in Figure 2. We find that most misdetection cases deviate from the ground truth by only a small number of pills ±1.

**Figure 3.**
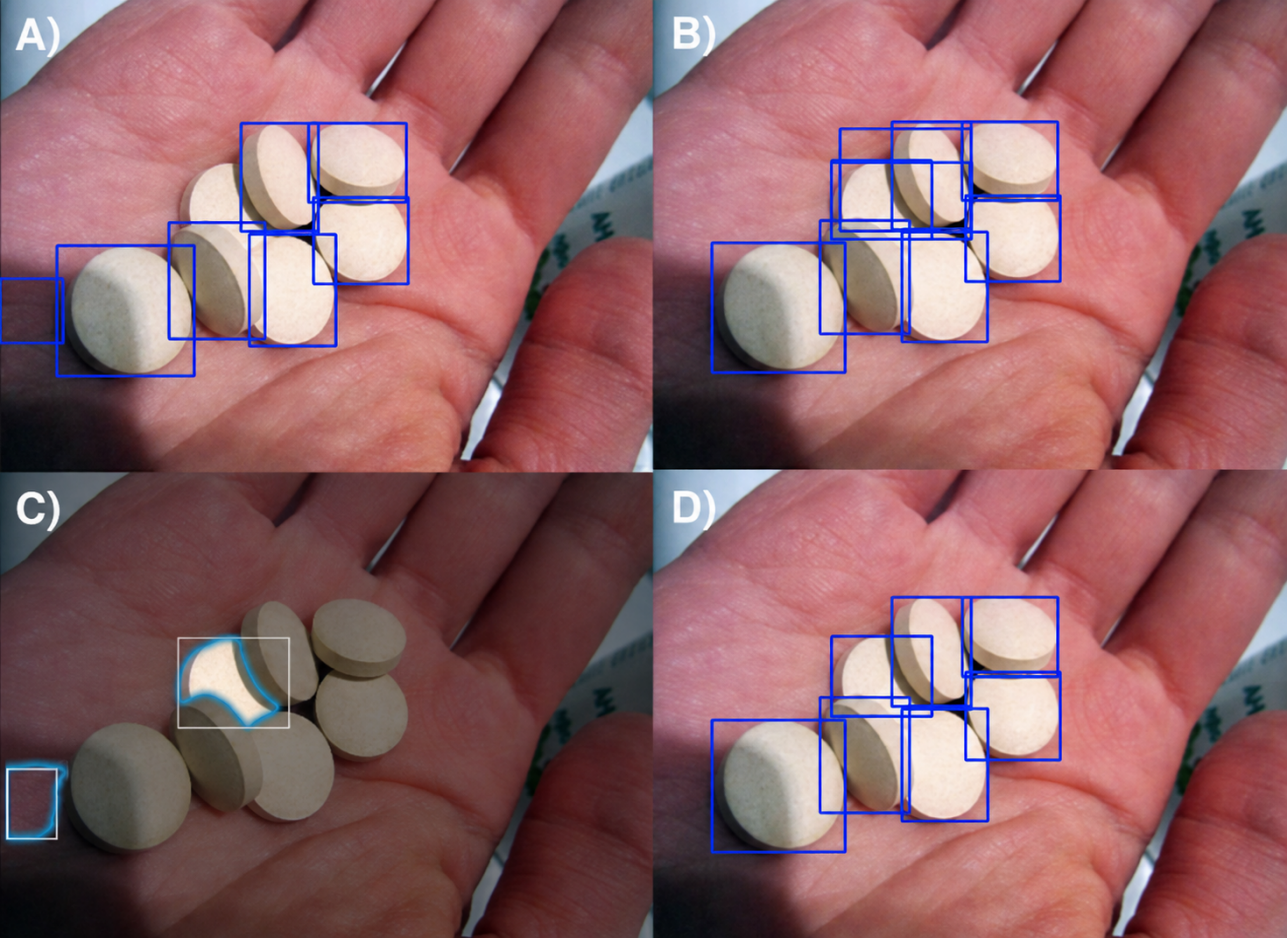
(**A**) Faster R-CNN detection results; (**B**) YOLOv12 detection results; (**C**) SAM segmentation results using bounding boxes as prompts; (**D**) Detection results from our improved multimodel framework. R-CNN: Faster Region-based Convolutional Neural Network; SAM: segment anything model; YOLOv12: You Only Look Once version 12.

Representative results are shown in Figure 3. Faster R-CNN frequently misclassifies background regions with pill-like appearance as pills and occasionally fails to detect all pills in the scene (Figure 3A). YOLOv12 reduces background false positives but tends to generate multiple overlapping bounding boxes for the same pill when pills are closely clustered, leading to over-counting errors as shown in Figure 3B. Figure 3C illustrates the segmentation results produced by SAM using detection bounding boxes as prompts, which provide improved boundary delineation but do not directly resolve redundancy or misclassification issues. By integrating individual detections from YOLOv12 and Faster R-CNN with SAM-based refinement and geometric validation, the proposed framework (Figure 3D) effectively suppresses redundant detections and removes spurious background regions, resulting in more accurate pill localization and counting.

### Longitudinal Medication Adherence Assessment

Adherence assessment examines whether we can automatically determine if the subject follows the prescription (ie, four pills per dose) from the image. As illustrated in Table 2, the proposed multimodel framework achieved an overall accuracy of 91.67%, with a sensitivity of 93.33% for detecting adherent cases and a specificity of 90.00% for identifying nonadherent cases. In comparison, we observe that YOLOv12 and Faster R-CNN show limited generalizability when evaluated in adherence simulation test on real-world datasets. This observation is consistent with prior findings showing that standard Faster R-CNN detectors often experience significant performance degradation under real-world domain shifts [32]. By incorporating the SAM foundation model trained on large-scale real-world data, the proposed framework demonstrates robustness across diverse real-world imaging conditions. Notably, the Faster R-CNN has much lower performance in our curated dataset than in Table 1, because it has limited generalizability to identify pills obtained in various scenarios, such as outdoor, office, restaurant, etc. This pilot study shows that the proposed framework can robustly support automated medication adherence assessment by accurately distinguishing compliant and noncompliant pill-taking behaviors in real-world imaging conditions.

**Table 2.** Adherence assessment performance on curated dataset.

Metrics	Proposed	YOLOv12^a^	Faster R-CNN^b^
Accuracy	0.917	0.600	0.183
Sensitivity	0.933	0.467	0.067
Specificity	0.900	0.733	0.300

a YOLOv12: You Only Look Once version 12.

b R-CNN: Region-based Convolutional Neural Network

**Figure 4.**
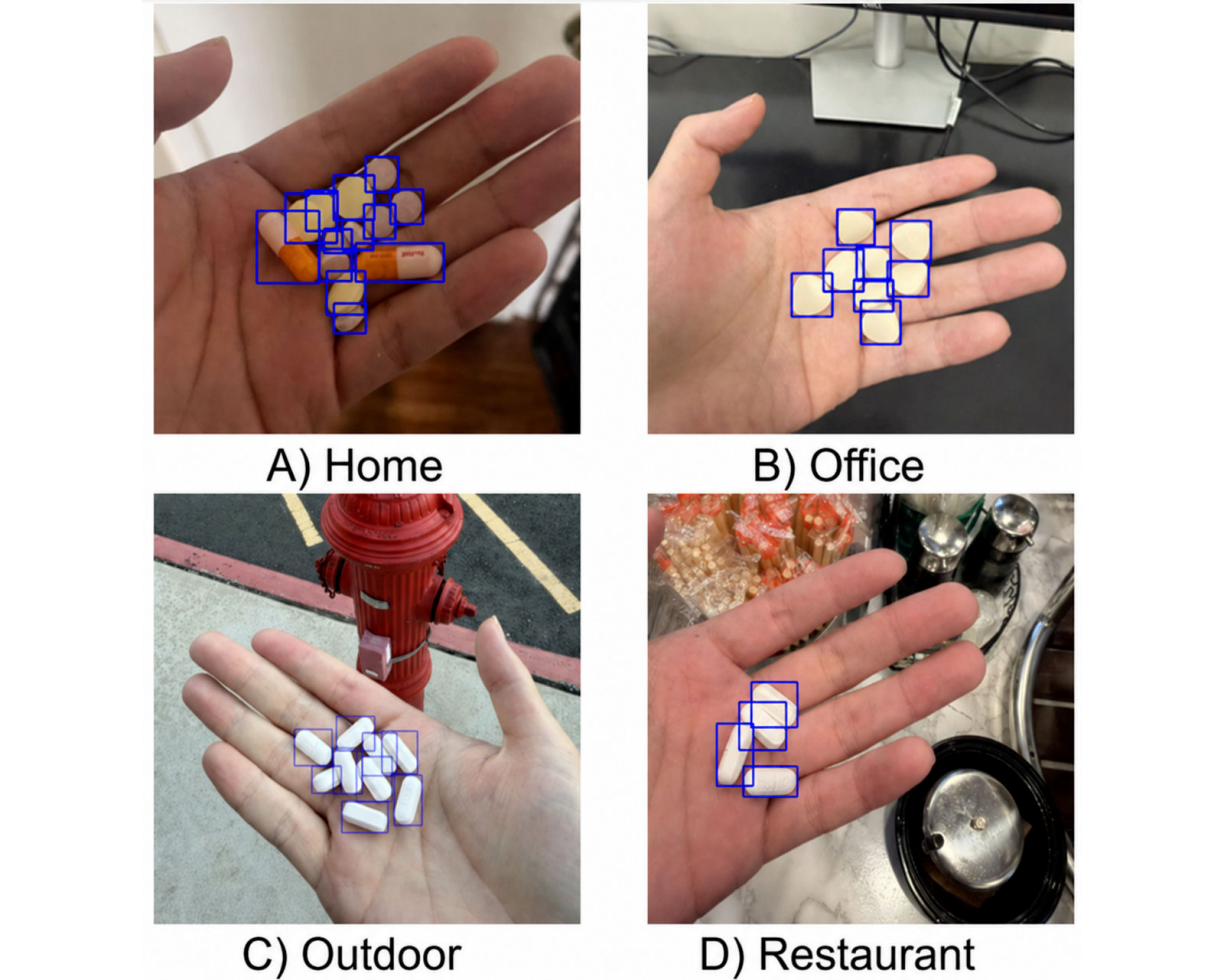
Representative examples of pill detection results in common real-world medication-taking environments, including (A) home, (B) office, (C) outdoor, and D) restaurant settings.

We additionally evaluated the proposed framework across common pill-taking environments that represent typical medication-taking scenarios. Pill images were collected in diverse locations frequently encountered in daily life, such as at home, office, outdoor, and restaurant. Those images cover scenarios that are dark (Figure 4A) and bright (Figure 4B). The shape of pills is also included in capsules (Figure 4A), round (Figure 4A), triangles (Figure 4B), and oblong (Figure 4C,D). As shown in Figure 4, our framework correctly identified number of pills among those scenarios. These experiments show the stability under realistic environmental variations beyond controlled test conditions. The results also show capability of detecting pills that are occluded. The proposed framework consistently maintained reliable detection and counting performance across these scenarios.

## Discussion

### Summary

This proof-of-concept study developed and evaluated a multimodel computer vision framework for pill detection and counting to support objective medication adherence assessment. The proposed framework achieved low pill-counting error and high dosage-level classification accuracy on both a public pill dataset and a curated longitudinal adherence simulation. These findings suggest that combining YOLOv12, Faster R-CNN, SAM-based segmentation refinement, redundancy removal, and geometric validation can improve still-image-based pill counting under daily-life imaging conditions.

The proposed approach addresses an important gap in medication adherence monitoring. Many existing adherence monitoring methods rely on indirect or proxy-based signals, such as self-report, pharmacy refill records, caregiver observation, electronic monitoring devices, and smart pill bottles. Although useful, these methods may not directly verify the visible pill count or dose-level correctness. In this study, the proposed framework outperformed the evaluated YOLOv12 and Faster R-CNN baselines, suggesting its potential for detecting both under-dosing and over-dosing scenarios.

### Limitations

This study has several limitations. First, the adherence scenario was simulated and did not involve real patient medication-taking behavior. Second, the curated dataset was modest in size and included limited pill appearances and imaging environments. Third, the current framework evaluates visible pill count but does not verify actual ingestion. Future studies should validate this approach in larger real-world human-subject cohorts and explore integration with longitudinal, privacy-preserving adherence monitoring workflows.

### Conclusions

This study presents a multimodel computer vision framework for objective, image-based medication adherence assessment. The results suggest that still-image-based pill detection and counting may serve as a useful component of scalable digital health tools for dose-level adherence monitoring. Larger real-world studies are needed to evaluate generalizability, usability, and clinical integration.

## Abbreviations

AI: Artificial Intelligence
IoU: intersection over union
MAE: Mean Absolute Error
MoE: Mixture of Experts
R-CNN: Region-based Convolutional Neural Network
SAM: Segment Anything Model
YOLOv12: You Only Look Once version 12
